# *Hes5*^+^ astrocytes potentiate primary afferent Aδ and C fiber-mediated excitatory synaptic transmission to spinal lamina I neurons

**DOI:** 10.1186/s13041-025-01212-y

**Published:** 2025-04-27

**Authors:** Itsuki Kagiyama, Sawako Uchiyama, Makoto Tsuda

**Affiliations:** 1 Department of Molecular and System Pharmacology, Graduate School of Pharmaceutical Sciences, Kyushu University, 3-1-1 Maidashi, Higashi-ku, Fukuoka, 812-8582 Japan; 2 Kyushu University Institute for Advanced Study, Fukuoka, Japan

**Keywords:** Astrocytes, Primary afferents, Spinal dorsal horn neurons, Excitatory synaptic transmission, N-methyl-D-aspartate receptor

## Abstract

Astrocytes are critical in regulating synaptic transmission in the central nervous system (CNS). The spinal dorsal horn (SDH) is a crucial region that processes and integrates somatosensory information from the periphery and transmits it to the brain. Our previous work demonstrated that stimulation of an astrocyte population in the SDH, characterized by the expression of hairy and enhancer of split 5 (*Hes5*), causes pain hypersensitivity. However, the mechanism by which *Hes5*^+^ astrocytes modulate synaptic transmission in the SDH remains unclear. In this study, using electrophysiological and cell type-specific functional manipulation approaches, we found that chemogenetic stimulation of *Hes5*^+^ SDH astrocytes enhanced Aδ and C fiber-mediated excitatory postsynaptic currents in lamina I neurons. A pharmacological blockade of the glycine binding site of N-methyl-D-aspartate (NMDA) receptors prevented the astrocytic enhancement. These findings suggest that *Hes5*^+^ astrocytes in the SDH enhance synaptic transmission from primary afferent nociceptors to lamina I neurons by potentiating NMDA receptor activity.

**Supplementary Information:**

The online version contains supplementary material available at 10.1186/s13041-025-01212-y.

Astrocytes in the central nervous system (CNS) are increasingly recognized as critical regulators of synaptic transmission [1, 2]. Growing evidence indicates the inter- and intra-regional diversity of astrocytes in gene expression, morphology, and function, attracting more attention to the role of individual subpopulations in CNS functions and diseases [2]. We recently identified a subpopulation of spinal cord astrocytes characterized by the expression of the transcription repressor hairy and enhancer of split 5 (*Hes5*) [3]. Within the spinal cord, the *Hes5*^+^ astrocyte subpopulation is selectively localized in the superficial laminae [3], a key area for receiving, processing, and integrating somatosensory (especially nociceptive) inputs from primary afferents before transmitting them to the brain [4]. We previously demonstrated that acute stimulation of *Hes5*^+^ spinal dorsal horn (SDH) astrocytes via G_q_ protein signaling induces behavioral pain hypersensitivity [3]. However, it remains unclear how *Hes5*^+^ astrocytes modulate nociceptive signaling from primary afferents to SDH neurons at the level of synaptic transmission.

This study investigated whether chemogenetic stimulation of *Hes5*^+^ SDH astrocytes affects primary afferent-derived synaptic transmission to lamina I neurons, which receive synaptic input from primary afferent nociceptors [4]. To express mutated G_q_ protein-coupled human M3 receptors (hM3Dq) in *Hes5*^+^ SDH astrocytes, we injected adeno-associated virus (AAV) vectors expressing hM3Dq and a hemagglutinin tag (HA) under the control of the FLEx switch system (AAV-flex[HA-hM3Dq]) into the SDH of *Hes5-CreERT2* mice (Fig. 1A; also see Additional file 1). Consistent with our previous findings [3], these tamoxifen-treated mice (*Hes5-CreERT2*; AAV-flex[HA-hM3Dq] mice) expressed hM3Dq (detected by HA immunostaining) in cells expressing the astrocyte markers glial fibrillary acidic protein (GFAP) and SRY-related high-mobility group box 9 (SOX9), but not cells expressing the neuronal marker (NeuN) and the microglia marker (IBA1) (Fig. 1B). Using spinal cord slices with the L4 dorsal root from *Hes5-CreERT2*; AAV-flex[HA-hM3Dq] mice, we performed whole-cell patch-clamp recordings of lamina I neurons (Fig. 1C). Electrical stimulation of the dorsal root at an intensity that can stimulate Aδ and C (Aδ/C) fibers [5] produced excitatory postsynaptic currents (EPSCs) in lamina I neurons (Fig. 1C). These evoked EPSCs were considered mono- and/or polysynaptic. The Aδ/C fiber-evoked mono/poly synaptic EPSCs did not exhibit failures on repetitive stimulation at 1 and 20 Hz, consistent with previous studies [5]. We found that application of the hM3Dq agonist deschloroclozapine (DCZ) to the spinal cord slices did not affect the amplitude of Aδ/C fiber-evoked monosynaptic EPSCs (Fig. 1D) but significantly increased the amplitude of polysynaptic EPSCs (Fig. 1E). To exclude the possibility that the observed potentiation was restricted to the *Hes5-CreERT2* mouse line, hM3Dq was expressed in SDH astrocytes (including *Hes5*^+^ astrocytes) of wild-type (WT) mice by intra-SDH injection of AAV-gfaABC_1_D-HA-hM3Dq (gfaABC_1_D: an astrocytic promoter) (Fig. 1F, G). DCZ applied to spinal cord slices from these mice similarly increased the amplitude of polysynaptic EPSCs in lamina I neurons evoked by electrical stimulation of Aδ/C fibers (Fig. 1H), supporting the conclusion that *Hes5*^+^ astrocytes enhance nociceptive polysynaptic transmission in the SDH.

**Fig. 1:**
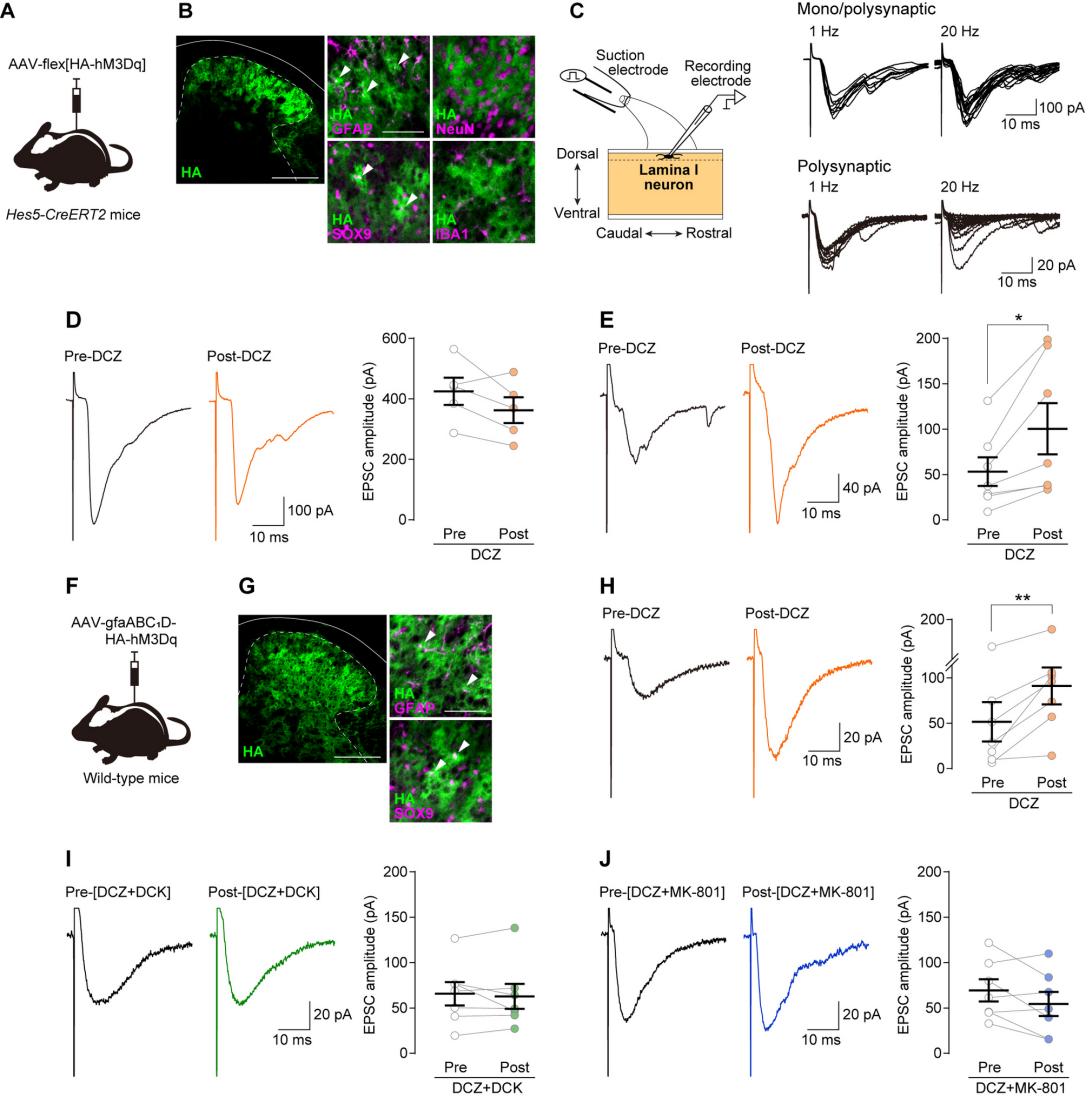
Chemogenetic stimulation of *Hes5*^+^ SDH astrocytes enhances Aδ/C fiber-mediated excitatory synaptic transmission to lamina I neurons. (**A**) Schematic illustration of transduction strategy of hM3Dq expression in *Hes5*^+^ SDH astrocytes. (**B**) Expression of hM3Dq (green; detected by HA immunostaining) in the SDH of *Hes5-CreERT2*; AAV-flex[HA-hM3Dq] mice. Dashed lines indicate the boundary between the gray and white matters. Double immunostaining of HA^+^ cells with cell type markers (magenta; GFAP, SOX9, NeuN, and IBA1). Arrowheads indicate GFAP^+^HA^+^ and SOX9^+^HA^+^ astrocytes. Scale bars: 200 μm (left) and 50 μm (right). (**C**) Schematic diagram of whole-cell recording in lamina I neurons using sagittal spinal cord slices with the L4 dorsal root from *Hes5-CreERT2*; AAV-flex[HA-hM3Dq] mice (left). Representative traces of Aδ/C fiber-evoked mono- and/or polysynaptic EPSCs in lamina I neurons (right). (**D**, **E**) Representative traces and quantitative analyses of the amplitudes of Aδ/C fiber-evoked mono/polysynaptic (**D**) and polysynaptic (**E**) EPSCs in lamina I neurons of *Hes5-CreERT2*; AAV-flex[HA-hM3Dq] mice before (Pre-DCZ) and after DCZ (5 µM) application (Post-DCZ) (*n* = 5 and 7 neurons, respectively). *P < 0.05. (**F**) Schematic illustration of transduction strategy of hM3Dq expression in SDH astrocytes of wild-type mice. (**G**) Expression of hM3Dq (green, detected by HA immunostaining) in the SDH at 3 weeks after microinjection of AAV-gfaABC_1_D-HA-hM3Dq in wild-type mice. Dashed lines indicate the boundary between the gray and white matters. Double immunostaining of HA^+^ cells with astrocyte markers (magenta; GFAP, and SOX9). Arrowheads indicate GFAP^+^HA^+^ and SOX9^+^HA^+^ astrocytes. Scale bars: 200 μm (left) and 50 μm (right). (**H**) Representative traces and amplitude of Aδ/C fiber-evoked polysynaptic EPSCs in lamina I neurons of WT mice with intra-SDH injection of AAV-gfaABC_1_D-HA-hM3Dq before (Pre-DCZ) and after DCZ (5 µM) application (Post-DCZ) (*n* = 7 neurons). **P < 0.01. (I, J) Effect of DCK (30 µM) and MK-801 (20 µM) on the DCZ-induced enhancement of Aδ/C fiber-evoked polysynaptic EPSCs in lamina I neurons of *Hes5-CreERT2*; AAV-flex[HA-hM3Dq] mice (*n* = 7 neurons, respectively). Data represent mean ± SEM

Astrocytes modulate synaptic transmission through various factors [1, 2]. In this study, we focused on the involvement of D-serine [1–3] as our previous study demonstrated that pain hypersensitivity induced by chemogenetic stimulation of *Hes5*^+^ SDH astrocytes was suppressed by 5,7-dichlorokynurenic acid (DCK), which antagonizes D-serine signaling via the glycine binding site on N-methyl-D-aspartate receptors (NMDARs). In spinal cord slices from *Hes5-CreERT2*; AAV-flex[HA-hM3Dq] mice, we found that the DCZ-enhanced amplitude of the Aδ/C fiber-evoked EPSCs was abolished by pretreating the slices with DCK (Fig. 1I) and MK-801 (an antagonist of NMDARs) (Fig. 1J). These findings suggest that NMDARs are involved in the astrocytic enhancement of synaptic transmission.

In this study, we demonstrated, for the first time, that chemogenetic stimulation of *Hes5*^+^ SDH astrocytes enhances excitatory synaptic transmission from primary afferent Aδ/C fibers to lamina I neurons. Astrocytes release various factors [1, 2], and our pharmacological data suggest that astrocytic enhancement likely involves D-serine signaling via the glycine binding site on NMDARs. This study did not measure D-serine release from astrocytes, and this needs further analyses using several tools (e.g., biosensors or microdialysis) in the future, but our hypothesis is supported by previous data showing astrocytic D-serine release from cerebellar slices [6] and a tendency towards its increased levels in spinal cord slices after chemogenetic stimulation of *Hes5*^+^ astrocytes [3]. In vivo, NMDAR-mediated SDH neuronal excitation is reduced by an antagonist of the glycine binding site of NMDARs [7]. Although the astrocytic potentiation was not observed in monosynaptic EPSCs, it may be due to our experimental conditions where the holding potential was set at -70 mV, where Mg^2+^ can block NMDARs (Additional file 2). Given that monosynaptic EPSCs are primarily mediated by AMPARs at -70 mV [8], it is conceivable that *Hes5*^+^ SDH astrocytes preferentially potentiate Aδ/C fiber-derived synaptic transmission to lamina I neurons via their enhancing effect on NMDAR activity in SDH interneurons (Additional file 2). In addition, further studies are needed to determine the roles of other cell types (e.g., microglia [9]) in *Hes5*^+^ astrocyte-derived enhancement of synaptic transmission in the SDH.

In the field of pain research, the mechanisms by which astrocytes modulate synaptic transmission have been primarily proposed by studies using pathological chronic pain models, in which the SDH and brain astrocytes respond and increase the expression of various genes, including proinflammatory cytokines and chemokines [10]. In the SDH, astrocytic proinflammatory cytokines potentiate EPSCs in lamina II neurons [11]. In the primary somatosensory cortex, astrocytes release factors such as thrombospondin, glypicans, and hevin, which remodel dendritic spines and reorganize synapses [12]. These astrocyte-modulating effects depend on the elevated expression levels of these factors in reactive astrocytes [10, 12]. This study shows that SDH astrocytes, even under physiological conditions, enhance excitatory synaptic transmission from Aδ/C fibers to lamina I neurons via the glycine binding site of NMDARs. Additionally, a similar acute modulatory effect of stimulated astrocytes in the SDH has been reported on inhibitory synaptic transmission [13]; specifically, optogenetic stimulation of SDH astrocytes reduces the excitability of inhibitory interneurons in lamina II via astrocytic ATP/adenosine signaling. Given that chemogenetic stimulation of *Hes5*^+^ astrocytes induces a similar effect [14], it is possible that activation of *Hes5*^+^ astrocytes can both enhance excitatory and reduce inhibitory synaptic transmission, resulting in enhanced net excitability of SDH neural circuits.

While this study did not directly link the observed synaptic changes to pain behavior, we have previously demonstrated that activation of hM3Dq in *Hes5*^+^ astrocytes in the SDH causes mechanical pain hypersensitivity via D-serine signaling on NMDARs [3]. Furthermore, among G_q_ protein-coupled receptors endogenously expressed in astrocytes and their ligands [15], *Hes5*^+^ SDH astrocytes have been shown to be activated by noradrenaline (NA) released from descending NAergic terminals via astrocytic α_1A_ receptors [3]. NAergic activation of *Hes5*^+^ SDH astrocytes enhances mechanical pain hypersensitivity (assessed via von Frey filaments), an effect that pretreatment with DCK prevents [3]. Given that behavioral response to these filaments partly involves nociceptors [16], activation of G_q_ protein-coupled receptors endogenously expressed in *Hes5*^+^ SDH astrocytes such as α_1A_ receptors may also enhance excitatory synaptic transmission from Aδ/C fibers to lamina I neurons and contribute to mechanical pain hypersensitivity.

## Electronic supplementary material

Below is the link to the electronic supplementary material.

Supplementary Material 1

Supplementary Material 2

## Data Availability

No datasets were generated or analysed during the current study.

## Abbreviations

AAV: Adeno-associated virus
CNS: Central nervous system
DCK: 5,7-dichlorokynurenic acid
DCZ: Deschloroclozapine
EPSC: Excitatory postsynaptic current
GFAP: Glial fibrillary acidic protein
HA: Hemagglutinin
Hes5: Hairy and enhancer of split 5
hM3Dq: The mutated G_q_ protein-coupled human M3 receptor
NA: Noradrenaline
NMDAR: N-methyl-D-aspartate receptor
SDH: Spinal dorsal horn
SOX9: SRY-related high-mobility group box 9
WT: Wild type
