# Supplementary material for: Hes5+ astrocytes potentiate primary afferent Aδ and C fiber-mediated excitatory synaptic transmission to spinal lamina I neurons
Source: Mol Brain. 2025 Apr 27;18:39. doi: 10.1186/s13041-025-01212-y (PMC12036120; doi:10.1186/s13041-025-01212-y)
Supplement: Supplementary file 1 — Supplementary Material 1 [file 13041_2025_1212_MOESM1_ESM.docx]

**Additional file 1**

**Materials and methods**

**Animals**

Male C57BL/6J mice (CLEA Japan), and male and female *Hes5-CreERT2* mice [Tg(*Hes5-cre/ERT2*)*^2Vtlr^*] (kindly provided by Prof. Verdon Taylor) [1] were used. For induction of Cre recombinase activity, *Hes5-CreERT2* mice were given an intraperitoneal (i.p.) injection of tamoxifen (#T5648, Sigma-Aldrich; 2 mg dissolved in 100 μl corn oil [#032-17016; Wako, Saitama, Japan]) once a day for 5–10 consecutive days [2, 3]. We used tamoxifen-injected mice for further analyses 7 days or more after the last tamoxifen injection. All mice used were 8–12 weeks of age at the start of each experiment and were housed at temperature and humidity ranges of 21–23°C and 40–60%, respectively, with a 12-h light-dark cycle. All animals were fed food and water ad libitum. All animals were housed in standard polycarbonate cages in groups of same-sex littermates. All animal experiments were conducted according to relevant national and international guidelines contained in the ‘Act on Welfare and Management of Animals’ (Ministry of Environment of Japan) and ‘Regulation of Laboratory Animals’ (Kyushu University) and under the protocols approved by the Institutional Animal Care and Use committee review panels at Kyushu University.

**Recombinant AAV (rAAV) vector production**

According to our previously described methods [2], the genes encoding hM3Dq (#45547; Addgene) were subcloned into the pENTR plasmid (Thermo Fisher Scientific). To produce AAV vectors, we inserted HA-hM3Dq into pZac2.1-gfaABC_1_D-WPRE plasmid. To produce the AAV vector for the Cre-switch system, vectors containing the promoter encoding EF1α were generated from pAAV-CA-FLEX (#38042; Addgene) by substituting the promoter. We then inserted hM3Dq (#45547; Addgene) into pAAV-EF1α-FLEX. rAAV vectors were produced from human embryonic kidney 293T (HEK293T) cells with triple transfection [pZac or pAAV, cis plasmid; pAAV2/5 (University of Pennsylvania Gene Therapy Program Vector Core) (#81070; Addgene), trans plasmid; pAd DeltaF6, adenoviral helper plasmid (University of Pennsylvania Gene Therapy Program Vector Core)] and purified by two cesium chloride density gradient purification steps. The vectors were dialyzed against phosphate-buffered saline (PBS; #041-20211; Wako, Saitama, Japan) containing 0.001% (v/v) Pluronic-F68 (#24040032; Thermo Fisher Scientific) using Vivaspin Turbo 15 100,000 MWCO (#VS15T41; Sartorius). The genome titer of rAAV was determined by Pico Green fluorometric reagent (#P7589; Thermo Fisher Scientific) following denaturation of the AAV particles. Vectors (AAV2/5-gfaABC_1_D-HA-hM3Dq-WPRE and AAV2/5-flex[HA-hM3Dq]-WPRE) were stored at −80°C until use.

**Intra-spinal dorsal horn (SDH) and injection of rAAV vectors**

Viral vector injection was performed in accordance with our previously described methods [2, 4]. Mice were deeply anesthetized by subcutaneous injection of ketamine (100 mg/kg) and xylazine (10 mg/kg). For intra-SDH injection, the skin was incised at Th11–L4 vertebrae, and custom-made clamps were attached to the caudal sites of the vertebral column. Paraspinal muscles around the left side of the interspace between Th13 and L1 vertebrae were removed, and the dura mater and arachnoid membrane were carefully incised using the tip of a 30-G needle to make a small window allowing a glass microcapillary to insert directly into the SDH. The microcapillary was inserted into the unilateral SDH (around 120–150 μm in depth from the surface of the dorsal root entry zone). rAAV solutions (approximately 500 nl) were injected using a Micro4 Micro Syringe Pump Controller (World Precision Instrument). After microinjection, the glass microcapillary was removed, the skin was sutured with 5-0 silk, and the mice were kept on a heating pad until recovery. We used virus-injected mice for further analyses 3 weeks or more after the AAV injection. The titers of viral vectors were as follows: AAV2/5-gfaABC_1_D-HA-hM3Dq-WPRE (injected to C57BL/6J (WT) mice), AAV2/5-EF1α-flex[HA-hM3Dq]-WPRE (injected to *Hes5-CreERT2* mice): 1.0 × 10^12^ genome copies (GC)/ml.

**Immunohistochemistry**

As described in our previous study [2], mice were deeply anesthetized with an i.p. injection of pentobarbital and transcardially perfused with PBS followed by ice-cold 4% paraformaldehyde (#162-16065; Wako, Saitama, Japan)/PBS. The transverse L4 segments of the spinal cord were removed, postfixed in the same fixative for 3 h at 4°C, and placed in 30% sucrose solution for 24hours at 4°C. After incubation, the tissues were embedded in OCT compound (#4583; Sakura Finetek Japan, Osaka, Japan) and stored at −25°C before use. Transverse spinal cord sections (30 μm) were incubated in blocking solution (3% normal goat serum [#S-1000; Vector Laboratories] or normal donkey serum [#017-000-121; Jackson ImmunoResearch]) for 2 h at room temperature and then incubated for 48 h at 4°C with primary antibodies: monoclonal rabbit anti-hemagglutinin-tag (HA; 1:1000; #3724; Cell Signaling); monoclonal rat anti-glial fibrillary acidic protein (GFAP; 1:2000; #13-0300; Invitrogen); polyclonal goat anti-SRY-related high-mobility group box 9 (SOX9; 1:1000; #AF3075; R&D Systems); monoclonal mouse anti-neuronal nuclei (NeuN; 1:1000; #ab104224; Abcam); polyclonal guinea pig anti-ionized calcium-binding adapter molecule 1 (IBA1; 1:2000; #234004; Synaptic systems). After incubation, tissue sections were washed and incubated for 3 h at room temperature with secondary antibodies (Alexa Fluor™ 488, 546; #A21206, A10036, and A11056; Thermo Fisher Scientific: Cy3; #706-165-148, and 712-165-153). Then, tissue sections were washed, slide mounted, and subsequently placed under coverslips with VECTASHIELD Hardmount (Vector Laboratories). Immunofluorescence images were obtained with confocal laser microscopy (LSM700; Carl Zeiss).

**Whole-cell patch-clamp recordings**

According to our previously described method[5] mice were deeply anesthetized with urethane (1.2–1.5 mg/kg, i.p.), and the lumbar spinal cord was removed and placed in a cold high-sucrose aCSF (250 mM sucrose, 2.5 mM KCl, 2 mM CaCl_2_, 2 mM MgCl_2_, 1.2 mM NaH_2_PO_4_, 25 mM NaHCO_3_, and 11 mM glucose). Parasagittal spinal cord slices with L4 dorsal root (250–300 µm thick) were made with a vibrating microtome (VT1200, Leica) and then the slices kept in oxygenated aCSF solution (125 mM NaCl, 2.5 mM KCl, 2 mM CaCl_2_, 1 mM MgCl_2_, 1.25 mM NaH_2_PO_4_, 26 mM NaHCO_3_, and 20 mM glucose) at room temperature (22–25°C) for at least 30 min. The spinal cord slice was then put into a recording chamber, where it was continuously superfused with aCSF solution at 25–28°C at a flow rate of 4–6 ml/min. Recordings were made with the Axopatch 700B amplifier and pCLAMP 10.4 acquisition software (Molecular Devices). Data were digitized with an analog-to-digital converter (Digidata 1550; Molecular Devices), stored on a personal computer with a data acquisition program (ClampeX version 10.4; Molecular Devices), and analyzed with a software package (Clampfit version 10.7; Molecular Devices). Excitatory postsynaptic currents (EPSCs) were recorded in the voltage-clamp mode at a holding potential (-70 mV). Patch pipettes were filled with an internal solution (125 mM K-gluconate, 10 mM KCl, 0.5 mM EGTA, 10 mM HEPES, 5 mM QX-314, 4 mM ATP-Mg, 0.3 mM NaGTP, 10 mM phosphocreatine, pH 7.28 adjusted with KOH). The pipette tip resistance was 6–10 MΩ. Whole-cell patch-clamp recordings were made from lamina I neurons (at a distance of < 20 μm from the border between the white and gray matters in the SDH[6]). The dorsal roots were stimulated with a suction electrode. Electrical stimuli (intensity, 1000 μA; duration, 0.1 ms) were applied to the dorsal root for stimulating Aδ and C fibers as described previously[7]. The Aδ/C fiber-evoked EPSCs that did not exhibit failures on repetitive stimulation at 1 and 20 Hz were analyzed as monosynaptic [7, 8]. In this study, we confirmed that the Aδ/C fiber-evoked polysynaptic EPSCs exhibited failures on repetitive stimulation at 1 and 20 Hz. The following drugs were used: deschloroclozapine (DCZ; 5 µM; #7193; Tocris Bioscience), 5,7-dichlorokynurenic acid (DCK; 30 µM; #131123-76-7; Tocris Bioscience), (+)-MK-801 hydrogen maleate (20 µM; #M107; Sigma-Aldrich). All drugs were dissolved in aCSF solution. DCZ was continuously superfused from 2 min before electrical stimulation. Bath application of DCK and MK-801 was continuously superfused from 2 min before DCZ application. Lamina I neurons with abnormally high amplitudes of spontaneous EPSCs before drug administration were excluded from subsequent experiments because it was difficult to precisely distinguish between these spontaneous EPSCs and the dorsal root stimulation-evoked polysynaptic EPSCs. The amplitude of evoked EPSCs were quantified using Clampfit version 10.7 (Molecular Devices).

**Statistical analysis**

Statistical analyses were performed using Prism 7 (GraphPad). All data are shown as the mean ± SEM. Statistical significance of differences was determined using paired *t*-test (Fig.1D, E, H, I and J). Differences were considered significant at *P* < 0.05.

**References**

1. Lugert S, Vogt M, Tchorz JS, Muller M, Giachino C, Taylor V. Homeostatic neurogenesis in the adult hippocampus does not involve amplification of Ascl1(high) intermediate progenitors. *Nat Commun*. **2012**;3:670.

2. Kohro Y, Matsuda T, Yoshihara K, Kohno K, Koga K, Katsuragi R, et al. Spinal astrocytes in superficial laminae gate brainstem descending control of mechanosensory hypersensitivity. *Nat Neurosci*. **2020**;23(11):1376-87.

3. Kawanabe-Kobayashi R, Uchiyama S, Yoshihara K, Kojima D, McHugh T, Hatada I, et al. Descending locus coeruleus noradrenergic signaling to spinal astrocyte subset is required for stress-induced pain facilitation. *bioRxiv*. **2024**:2024.11.14.623627.

4. Kohro Y, Sakaguchi E, Tashima R, Tozaki-Saitoh H, Okano H, Inoue K, et al. A new minimally-invasive method for microinjection into the mouse spinal dorsal horn. *Sci Rep*. **2015**;5:14306.

5. Uchiyama S, Yoshihara K, Kawanabe R, Hatada I, Koga K, Tsuda M. Stress-induced antinociception to noxious heat requires alpha(1A)-adrenaline receptors of spinal inhibitory neurons in mice. *Mol Brain*. **2022**;15(1):6.

6. Dougherty KJ, Sawchuk MA, Hochman S. Properties of mouse spinal lamina I GABAergic interneurons. *J Neurophysiol*. **2005**;94(5):3221-7.

7. Baba H, Ji RR, Kohno T, Moore KA, Ataka T, Wakai A, et al. Removal of GABAergic inhibition facilitates polysynaptic A fiber-mediated excitatory transmission to the superficial spinal dorsal horn. *Mol Cell Neurosci*. **2003**;24(3):818-30.

8. Nakatsuka T, Ataka T, Kumamoto E, Tamaki T, Yoshimura M. Alteration in synaptic inputs through C-afferent fibers to substantia gelatinosa neurons of the rat spinal dorsal horn during postnatal development. *Neuroscience*. **2000**;99(3):549-56.
