# Supplementary material for: Hes5+ astrocytes potentiate primary afferent Aδ and C fiber-mediated excitatory synaptic transmission to spinal lamina I neurons
Source: Mol Brain. 2025 Apr 27;18:39. doi: 10.1186/s13041-025-01212-y (PMC12036120; doi:10.1186/s13041-025-01212-y)
Supplement: Supplementary file 2 — Supplementary Material 2 [file 13041_2025_1212_MOESM2_ESM.docx]

**Additional file 2**


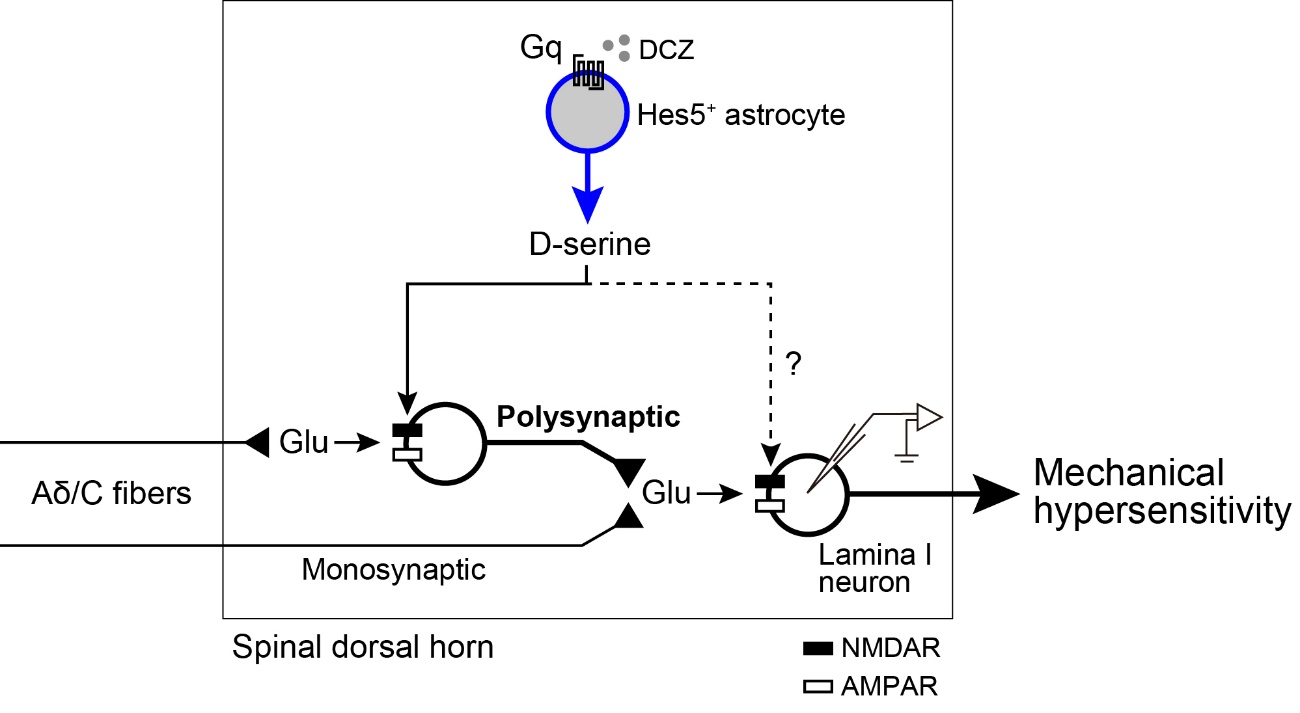


**Schematic illustration of the possible monosynaptic and polysynaptic neural circuits from primary afferent** **Aδ/C fibers to lamina I neurons**

Nociceptive information via primary afferent Aδ/C fibers is transmitted mono- and polysynaptically to lamina I neurons (recorded in this study) in the spinal dorsal horn (SDH). Chemogenetic stimulation of *Hes5*^+^ astrocytes enhances polysynaptic transmission to lamina Ⅰ neurons by potentiating NMDAR activity via D-serine signaling. Although such effect was not observed in monosynaptic transmission to lamina I neurons in this study, this may be due to our experimental conditions where the holding potential of recorded cells was set at -70 mV, where Mg^2+^ can block NMDARs. Given that chemogenetic stimulation of *Hes5*^+^ astrocytes in the SDH induced mechanical hypersensitivity via NMDARs and that the effect was suppressed by DCK [1], the *Hes5*^+^ astrocyte-derived enhancement of excitatory synaptic transmission may be involved in mechanical hypersensitivity.

**References**

1. Kohro Y, Matsuda T, Yoshihara K, Kohno K, Koga K, Katsuragi R, et al. Spinal astrocytes in superficial laminae gate brainstem descending control of mechanosensory hypersensitivity. *Nat Neurosci*. **2020**;23(11):1376-87.
